# From Simulation to Sustainability: The Mediating Role of Clinical Self-Efficacy Among Undergraduate Healthcare Students

**DOI:** 10.3390/ejihpe16060075

**Published:** 2026-05-26

**Authors:** Waleed El-Sayed Mohammed Hemaida, Ekram Mohammed Gomaa Geenedy, Mohamed Sayed Abdellatif, Mohamed Ali Nemt-allah

**Affiliations:** 1 Department of Nursing, College of Applied Medical Sciences, Prince Sattam Bin Abdulaziz University, Wadi Addawasir 11991, Saudi Arabia; w.hemaida@psau.edu.sa; 2 Adult Health Nursing Department, Critical Care Nursing, Faculty of Nursing, Capital University, Helwan 11731, Egypt; ekram_geneedy@nursing.helwan.edu.eg; 3 Department of Psychology, College of Education in Al-Kharj, Prince Sattam bin Abdulaziz University, Al-Kharj 11942, Saudi Arabia; m.heby@psau.edu.sa; 4 Educational Psychology and Statistics Department, Faculty of Education, Al-Azhar University, Tafhna Al-Ashraf 35822, Egypt

**Keywords:** simulation-based learning, clinical performance self-efficacy, sustainability attitudes, nursing education, mediation analysis, social cognitive theory

## Abstract

Despite growing recognition that nurses must be equipped with sustainability competencies to address climate-related health challenges, the psychological mechanisms through which nursing education fosters sustainability attitudes are not yet fully understood. This study examined the mediating role of clinical performance self-efficacy in the relationship between simulation-based learning quality and sustainability attitudes among undergraduate nursing students. A cross-sectional correlational design was employed with a main sample of 679 nursing students from four Egyptian universities. Data were collected using the CHEST, SECP Scale, and SANS_2. Mediation analysis used Hayes’ PROCESS macro with 5000 bootstrap resamples. Simulation-based learning quality significantly predicted both self-efficacy (β* = 0.772) and sustainability attitudes (β* = 0.613). Self-efficacy partially mediated this relationship, accounting for 68.34% of the total effect (indirect β* = 0.419, Boot 95% CI [0.343, 0.494]). Nursing educators should design simulation curricula that deliberately cultivate self-efficacy while embedding sustainability content, producing clinically competent and environmentally responsible graduates.

## 1. Introduction

The integration of sustainability attitudes into modern nursing education has become an urgent global imperative, driven by the recognition that healthcare systems are simultaneously contributors to and victims of environmental degradation (Walpole et al., 2019). As healthcare accounts for approximately 4–5% of global greenhouse gas emissions, nurses—as frontline decision-makers—must be equipped with the knowledge to understand how climate change, pollution, and ecological disruption directly affect population health outcomes (Álvarez-Nieto et al., 2021; Hendry et al., 2025). Beyond environmental literacy, sustainability education cultivates competencies in resource management, waste reduction, and green clinical practice (Attia et al., 2025; Hamid & Ibrahim, 2025). Furthermore, nursing education framed around sustainable development positions nurses as advocates for health equity, social justice, and the United Nations Sustainable Development Goals, underscoring the profound long-term social consequences embedded within every dimension of healthcare delivery (Boakye et al., 2024; Levett-Jones et al., 2024).

Simulation-based learning has emerged as a transformative pedagogical strategy in nursing education, broadly defined as an educational technique that recreates realistic clinical situations within a safe, structured environment where students can practice, make errors, and refine their skills without endangering real patients (Kim et al., 2016; Miller, 2023). High-quality simulation is characterized by clear learning objectives, pre-briefing protocols, realistic scenario-based cases, and structured debriefing that promotes reflective practice and bridges the theory–practice gap (Alsadi et al., 2025; Daneshfar & Moonaghi, 2025; Mishra & Trivedi, 2023). Evidence consistently demonstrates that such environments significantly enhance students’ clinical competence, decision-making, self-confidence, and professional identity across diverse nursing contexts (Alharbi et al., 2024; Anbari & Kerari, 2025; Tong et al., 2024). Crucially, because ethical constraints limit direct student exposure to high-risk clinical situations, simulation serves as an indispensable substitute that maintains educational rigor while upholding patient safety standards (Kaldheim et al., 2022; Kingston et al., 2025).

Cultivating sustainability attitudes in nursing students requires purposeful instructional approaches that embed environmental values within authentic professional contexts. Simulation-based learning represents one such approach, shaping sustainability orientations by integrating resource conservation and ethical decision-making within realistic clinical scenarios (Mitchell et al., 2025; Wang et al., 2025). Specifically, well-designed scenarios incorporating waste management, consumable stewardship, and climate-health intersections have demonstrated significant improvements in students’ environmental awareness and pro-sustainability orientations (Álvarez-Nieto et al., 2022; Richardson & Grose, 2017). Active engagement with ethical dilemmas—such as choosing between single-use and reusable materials under resource scarcity—normalizes sustainability as a core professional responsibility (Mitchell et al., 2025; Tejedor et al., 2019). Furthermore, structured debriefing and clearly defined learning outcomes within high-quality simulations facilitate the transfer of sustainability-conscious attitudes into intended clinical practice behaviors (Anbari & Kerari, 2025; Daneshfar & Moonaghi, 2025; Wang et al., 2025).

Grounded in Bandura’s self-efficacy theory, clinical performance self-efficacy in nursing students is defined as their situation-specific belief in their capability to organize, execute, and successfully complete required nursing tasks and exercise sound clinical judgment within real or simulated patient care environments (Barkhordari-Sharifabad et al., 2025; Momeni et al., 2025). This construct encompasses students’ confidence in performing discrete clinical tasks—including physical examination, medication administration, clinical handover, and patient assessment—as well as their capacity to make independent, informed decisions throughout the nursing process (Albooghobeish et al., 2023; Farzaneh et al., 2023; Zeyrek & Fidan, 2025). Distinguished from general academic confidence, clinical performance self-efficacy is inherently context-specific, fluctuating in response to educational experiences, reflective capacity, and the quality of clinical training environments encountered during preparation (Alrashidi et al., 2023; Gheisari et al., 2025; Olaussen et al., 2025). Strengthening this belief is increasingly recognized as foundational to producing competent, confident, and professionally resilient nursing graduates (Albaqawi et al., 2025; Chow et al., 2023).

Bandura’s Social Cognitive Theory (SCT) provides the foundational framework for understanding how educational environments shape professional beliefs and behaviors (Bandura, 1986; Lavoie et al., 2017). Central to SCT is triadic reciprocal determinism, wherein environmental inputs, personal cognitive beliefs, and behavioral intentions mutually influence one another (Hamann et al., 2023; Zhang et al., 2025). Within nursing education, SCT positions mastery experiences, vicarious learning, and constructive feedback as the primary sources of self-efficacy beliefs, which subsequently shape students’ professional intentions and value orientations (Erfanian et al., 2024; Wille et al., 2025). This framework clarifies how simulation environments, clinical self-efficacy, and sustainability attitudes are theoretically interconnected throughout this study (Hussain et al., 2021; Kaldheim et al., 2021).

Clinical performance self-efficacy functions as a vital internal motivator in nursing students by simultaneously attenuating anxiety and sustaining the persistent effort required to internalize complex professional values (Albaqawi et al., 2025; Momeni et al., 2025). Rooted in Bandura’s theoretical framework, students with stronger self-efficacy demonstrate greater resilience under clinical demands, reduced psychological distress, and diminished burnout, thereby preserving the cognitive and emotional resources necessary for value-driven professional engagement (Ozsaker et al., 2025; Xu et al., 2023; Yurt & Hayli, 2025; Zhu et al., 2023). Critically, elevated self-efficacy is associated with heightened internal motivation, goal persistence, and deeper professional identity formation—mechanisms that directly support the adoption of future-oriented values such as environmental sustainability and climate-responsible practice (Eweida et al., 2025; Jun & Lee, 2016; Zhao et al., 2023). By buffering the anxiety inherent in demanding clinical training, self-efficacy creates the psychological space for nursing students to meaningfully engage with and sustain complex sustainability-oriented attitudes beyond immediate educational contexts (Kassabry, 2023; Panahi et al., 2025; Sahebi & Barkhordari-Sharifabad, 2023).

High-quality simulation-based learning enhances clinical performance self-efficacy primarily through the engineering of structured mastery experiences, wherein repeated successful practice of realistic clinical tasks directly reinforces students’ belief in their own competence (Alrashidi et al., 2023; Takashiki et al., 2022). Consistent with Bandura’s self-efficacy theory, simulation environments facilitate deliberate, feedback-rich practice of complex procedures—from medication administration to emergency management—enabling students to progressively achieve independence on clearly defined performance benchmarks (Guerrero et al., 2024; La Cerra et al., 2019; Ozata & Dinc, 2025). The psychologically safe, error-tolerant nature of simulation ensures that corrected mistakes are encoded as genuine capability development rather than failure, amplifying the self-efficacy gains derived from each successful performance encounter (Anbari & Kerari, 2025; Daneshfar & Moonaghi, 2025; Ton et al., 2024). Systematic evidence further confirms that high-fidelity simulation produces significant, sustained gains in self-efficacy that positively correlate with objective clinical performance outcomes over time (Jawabreh et al., 2025; Ma et al., 2024; Offiah et al., 2019).

The present study examined the mediating role of clinical performance self-efficacy in the relationship between simulation-based learning quality and sustainability attitudes among Egyptian nursing students. While prior research has independently linked simulation quality to self-efficacy and self-efficacy to professional value adoption, these studies share notable limitations: many rely on small, single-institution samples (Kassabry, 2023; Kaldheim et al., 2021); are confined to Western educational contexts, limiting cross-cultural generalizability (Álvarez-Nieto et al., 2022); and lack integrative mediation frameworks that test the full pathway simultaneously. No study has explicitly unified these pathways within a single mediation model targeting sustainability attitudes. The present study therefore addresses these gaps by employing a large multi-university sample, an Arabic-adapted instrumentation protocol, and rigorous bootstrapped mediation analysis to provide the first integrated empirical test of this psychological mechanism. Grounded in SCT, three research questions guided the investigation: the nature and strength of relationships among constructs, whether self-efficacy significantly mediates the simulation–sustainability relationship, and what proportion of the total effect operates through this indirect pathway.

## 2. Materials and Methods

### 2.1. Study Design

This study employed a cross-sectional, correlational design. Data were collected via a structured self-report questionnaire distributed through Google Forms over four weeks (28 September–12 October 2025). The questionnaire was distributed to 1050 eligible students across the four universities; 724 responses were received (response rate = 68.95%), of which 45 were excluded due to incomplete data, yielding final samples of N = 645 (psychometric) and N = 679 (main). Prior to data collection, official permissions were obtained from the administrative authorities of each participating university, and ethical approval was granted by the Research Ethics Committee of the Faculty of Education, Al-Azhar University (Ref. No. EDU-REC-2025-008).

### 2.2. Participants

Participants were undergraduate nursing students recruited from Zagazig University, Beni-Suef University, Kafr El-Sheikh University, and Al-Azhar University using convenience sampling. The total enrolled nursing student population across the four universities was approximately 3200 students. Convenience sampling was employed, given the practical constraints of online data collection; students were recruited through faculty-distributed links during regular academic activities. This approach was justified by its feasibility for multi-site recruitment within the designated timeframe. Two independent samples were drawn: a psychometric sample (N = 645) for scale validation and a main sample (N = 679) for structural analyses. The mean age of the psychometric sample was 20.40 years (SD = 1.25), and that of the main sample was 20.47 years (SD = 1.29). Detailed demographic characteristics for both samples are presented in Table 1.

### 2.3. Measures

The Comprehensive Healthcare Education Simulation Tool (CHEST; Al-Hassan et al., 2025) assessed students’ perceptions of simulation-based learning quality across six dimensions: Environment and Resources, Reflect and Support, Cultural Relevance, Skills and Confidence, Engagement, and Motivation (40 items total). A representative item is “The simulation lab at our institution is well-equipped with modern and functional equipment.” Items are rated on a five-point Likert scale (1 = Strongly Disagree to 5 = Strongly Agree). CFA supported adequate model fit (χ^2^/df = 1.286, RMSEA = 0.021, CFI = 0.982, GFI = 0.932), and McDonald’s ω ranged from 0.856 to 0.905 across subscales (total ω = 0.911).

The Self-Efficacy in Clinical Performance (SECP) Scale (Cheraghi et al., 2009) measured confidence in executing clinical nursing tasks across four dimensions: Assessment, Diagnosis and Planning, Implementation, and Evaluation (37 items total). A representative item is “I am confident that in the clinical setting, I can collect significance data by physical assessment.” Each item is rated on a 0–100 scale (0 = complete lack of confidence; 100 = complete confidence). CFA indicated acceptable fit (χ^2^/df = 2.271, RMSEA = 0.044, CFI = 0.949, GFI = 0.843), with Cronbach’s α ranging from 0.844 to 0.943 and total ω = 0.948.

The Sustainability Attitudes in Nursing Survey (SANS_2; Richardson et al., 2016) assessed attitudes toward sustainability and climate change in nursing practice via five unidimensional items rated on a seven-point Likert scale (1 = Strongly Disagree to 7 = Strongly Agree). A representative item is “Climate change is an important issue for nursing.” CFA confirmed an excellent fit (χ^2^/df = 0.966, RMSEA = 0.030, CFI = 0.999, GFI = 0.997), with Cronbach’s α = 0.834 and McDonald’s ω = 0.834.

### 2.4. Translation Procedure

All three instruments were originally developed in English. To ensure linguistic equivalence for Arabic-speaking participants, the scales were subjected to a rigorous forward-backward translation process carried out by three bilingual experts with relevant subject-matter expertise. Discrepancies between translation versions were resolved through consensus discussion until conceptual equivalence was achieved.

### 2.5. Statistical Analysis

Data were analyzed using IBM SPSS Statistics version 27. To test the hypothesized mediation model, Hayes’ PROCESS macro (Model 4) was applied to the main sample, with bootstrapping based on 5000 resamples and 95% bias-corrected confidence intervals (CIs). The total, direct, and indirect effects of simulation-based learning quality (X) on sustainability attitudes (Y) through clinical performance self-efficacy (M) were estimated and interpreted based on the significance of the bootstrapped CIs.

Prior to multivariate analyses, key statistical assumptions were verified. Univariate normality was assessed via skewness and kurtosis indices, with all values falling within accepted thresholds (skewness < 2; kurtosis < 7; Curran et al., 1996). Linearity was confirmed through visual inspection of scatterplots between all variable pairs. Multicollinearity was evaluated using the Variance Inflation Factor (VIF) statistics; all VIF values were below 3.0, indicating no problematic collinearity. To address common method bias, Harman’s single-factor test was conducted; the single extracted factor accounted for 31.4% of total variance, falling below the 50% threshold, suggesting common method variance was not a substantial concern (Podsakoff et al., 2003).

## 3. Results

This section presents the findings from descriptive, correlational, and mediation analyses conducted on a main sample of 679 undergraduate nursing students (N = 679). Results are organized into four subsections: descriptive statistics, bivariate correlations, path coefficients of the mediation model, and the decomposition of total, direct, and indirect effects.

### 3.1. Descriptive Statistics

Descriptive statistics for all study variables are reported in Table 2. Regarding the CHEST, subscale means ranged from 19.73 (SD = 2.63) for Skills and Confidence to 41.27 (SD = 4.72) for Motivation, with a total CHEST mean of 163.42 (SD = 18.87) out of a maximum of 200. The Environment and Resources subscale yielded a mean of 20.64 (SD = 2.41) out of a possible 25, suggesting that students perceived their simulation environments as moderately well-resourced. Similarly, the Reflect and Support subscale mean of 20.98 (SD = 2.49) indicates a moderately favorable perception of reflective and supervisory support within simulation experiences. The SECP total mean was 2463.46 (SD = 464.14), and SANS_2 yielded a mean of 28.12 (SD = 4.79) out of 35. All skewness and kurtosis values fell within accepted thresholds, supporting approximate univariate normality (Curran et al., 1996; West et al., 1995).

### 3.2. Bivariate Correlations

Prior to testing the mediation model, Pearson bivariate correlations were examined among all study variables to confirm the preconditions for mediation analysis. As presented in Table 3, all correlations were statistically significant at the *p* < 0.001 level. The total CHEST score was positively and strongly correlated with total SECP (r = 0.772) and moderately correlated with SANS_2 (r = 0.613). Total SECP demonstrated a moderate-to-strong positive correlation with SANS_2 (r = 0.692). Among CHEST subscales, correlations with SANS_2 ranged from r = 0.548 (ER) to r = 0.576 (SC). Among SECP subscales, correlations with SANS_2 ranged from r = 0.646 (EVA) to r = 0.701 (IMP). Intercorrelations among SECP subscales were uniformly high (ranging from r = 0.910 to r = 0.946), reflecting the strong coherence of the clinical self-efficacy construct. These patterns confirm that the basic prerequisites for mediation—significant associations between the predictor, mediator, and outcome—were satisfactorily met.

### 3.3. Mediation Analysis

The regression of SECP on CHEST (Path a) yielded a significant positive coefficient (B = 18.993, SE = 0.600, t = 31.635, *p* < 0.001; β* = 0.772). In the full model, SECP (Path b) exerted a significant positive effect on sustainability attitudes (B = 0.006, SE = 0.004, t = 12.586, *p* < 0.001; β* = 0.542). The direct effect of CHEST on SANS_2, controlling for SECP (Path c’), remained statistically significant (B = 0.049, SE = 0.011, t = 4.513, *p* < 0.001; β* = 0.194), though considerably attenuated relative to the total effect. The overall model explained 49.42% of the variance in SANS_2 (R^2^ = 0.494, F(2, 676) = 330.23, *p* < 0.001). The results of the mediation analysis are presented in Table 4. 

### 3.4. Decomposition of Effects

The total effect of CHEST on SANS_2 was positive and significant (B = 0.155, β* = 0.613, 95% CI [0.1403, 0.1706]). When SECP was introduced as a mediator, the direct effect was attenuated to B = 0.049 (β* = 0.194, 95% CI [0.0278, 0.0707]), representing 31.73% of the total effect. The indirect effect through SECP was statistically significant (B = 0.106, β* = 0.419, Boot 95% CI [0.0858, 0.1270]), as the bootstrap confidence interval excluded zero, accounting for 68.34% of the total effect. Both direct and indirect effects were significant, confirming partial mediation. These findings underscore self-efficacy as the dominant motivational bridge between high-quality simulation experiences and pro-sustainability professional orientations. These total, direct, and indirect effects are summarized in Table 5.

**Figure 1:**
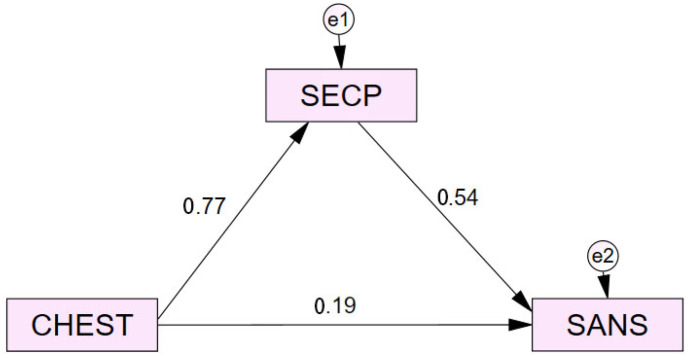
Mediation model: standardized path coefficients for the relationship between CHEST, SECP, and SANS_2.

The hypothesized mediation model and its associated standardized path coefficients are visually depicted in Figure 1. The figure illustrates the three core pathways: Path a (CHEST → SECP; β* = 0.772), Path b (SECP → SANS_2; β* = 0.542), and the attenuated direct Path c’ (CHEST → SANS_2; β* = 0.194), alongside the dominant indirect effect (β* = 0.419) that accounts for the majority of the total effect.

Collectively, the findings provide robust empirical support for the proposed mediation model, demonstrating that simulation-based learning quality exerts both a direct influence and a substantially larger indirect influence—mediated through clinical performance self-efficacy—on nursing students’ sustainability attitudes. The partial mediation pattern confirms that self-efficacy functions as the primary psychological mechanism translating high-quality simulation experiences into pro-sustainability professional orientations, accounting for over two-thirds of the total effect. These results underscore the theoretical coherence of the Social Cognitive Theory framework as applied to sustainability education in nursing and highlight the practical importance of designing simulation experiences that deliberately cultivate students’ clinical self-efficacy beliefs.

## 4. Discussion

The present study examined the mediating role of clinical performance self-efficacy in the relationship between simulation-based learning quality and sustainability attitudes among Egyptian nursing students. Results revealed that simulation-based learning quality was significantly positively associated with sustainability attitudes (β* = 0.613), with clinical performance self-efficacy mediating 68.34% of this effect. The direct effect of simulation quality on sustainability attitudes remained significant but substantially attenuated (β* = 0.194) after accounting for self-efficacy, confirming a partial mediation pattern. These findings indicate that high-quality simulation experiences are not solely associated with sustainability attitudes through direct instructional exposure; rather, they are primarily related to students’ belief in their clinical competence, which in turn is associated with the motivational and cognitive capacity to internalize complex professional values such as sustainability.

The present findings align with prior evidence and invite theoretical reintegration within Bandura’s SCT framework. Theoretically, this study makes a novel contribution by extending SCT’s triadic reciprocal determinism to the underexplored intersection of simulation pedagogy and sustainability education—a domain where integrated empirical frameworks have been notably absent. Prior studies examined simulation–self-efficacy and self-efficacy–professional values linkages in isolation, within predominantly Western, single-institution contexts (Álvarez-Nieto et al., 2022; Kaldheim et al., 2021). The present study advances the field by providing the first empirically integrated mediation model within a large, multi-university, non-Western sample, demonstrating that SCT’s motivational mechanisms operate cross-culturally and extend meaningfully into sustainability attitude formation—thereby offering a theoretically grounded blueprint for sustainability-conscious simulation curriculum design.

The partial mediation pattern warrants theoretical elaboration. Within SCT’s triadic reciprocal determinism, the significant residual direct association between simulation quality and sustainability attitudes suggests that simulation environments influence sustainability orientations through additional pathways beyond self-efficacy alone. Specifically, direct exposure to sustainability-themed scenarios may activate normative professional values independently of competence beliefs, consistent with values-based learning theories (Tejedor et al., 2019). Furthermore, variables such as reflective capacity, professional identity, and institutional sustainability culture may represent complementary mediating mechanisms accounting for the remaining variance (Barkhordari-Sharifabad et al., 2025; Boakye et al., 2024). Faculty role modeling during debriefing may also directly reinforce sustainability orientations without necessarily operating through self-efficacy pathways, suggesting a theoretically richer mediation landscape than the present model captures.

The findings carry meaningful implications for nursing educators, curriculum designers, and healthcare institutions. Simulation programs should be deliberately engineered not only to transmit clinical skills but also to cultivate robust self-efficacy beliefs through structured mastery experiences, constructive debriefing, and progressive performance benchmarks, as these mechanisms appear to be the primary conduit through which sustainability attitudes are formed (Anbari & Kerari, 2025; Daneshfar & Moonaghi, 2025). Integrating explicit sustainability content—including resource stewardship, waste management, and climate–health intersections—within simulation scenarios would further amplify both self-efficacy and sustainability orientations simultaneously. At the institutional level, investment in high-fidelity simulation infrastructure and faculty development in sustainability-conscious pedagogy represents a strategically efficient approach to producing nursing graduates who are both clinically confident and environmentally responsible, two competencies increasingly demanded by modern healthcare systems.

Several limitations should be considered. The cross-sectional design precludes causal inference; directionality cannot be empirically verified without longitudinal data. The study was conducted across four Egyptian universities, potentially limiting broader generalizability. Reliance on self-report measures introduces common method bias and social desirability responding. The SANS_2’s five-item structure may not fully capture the breadth of sustainability competencies relevant to nursing. Additionally, analyses were conducted using composite total scores rather than subscale-level scores, which may have obscured differential associations between specific simulation dimensions and self-efficacy or sustainability subdimensions. Furthermore, the non-use of structural equation modeling (SEM) represents a methodological constraint, as SEM would have enabled simultaneous estimation of measurement error and structural pathways, potentially yielding more precise parameter estimates than the regression-based mediation approach employed.

Future research should employ longitudinal or experimental designs to establish the causal directionality of the proposed pathways and assess whether self-efficacy gains from simulation durably predict sustainability behaviors in actual clinical practice settings. Comparative studies across diverse national and cultural contexts would strengthen the generalizability of the mediation model. Researchers should consider incorporating objective measures of clinical performance and behavioral sustainability outcomes alongside self-report instruments to reduce reliance on single-method assessment. Mediating and moderating variables beyond self-efficacy—including reflective capacity, professional identity, institutional sustainability culture, and faculty role modeling—warrant investigation as additional mechanisms. Intervention studies explicitly embedding sustainability content within structured simulation curricula, combined with rigorous debriefing protocols, would provide valuable experimental evidence for the proposed pathway.

## 5. Conclusions

This study provides robust empirical evidence that clinical performance self-efficacy functions as the dominant psychological mechanism through which simulation-based learning quality shapes nursing students’ sustainability attitudes, accounting for over two-thirds of the total effect within a partial mediation framework. These findings extend Social Cognitive Theory to the intersection of simulation pedagogy and sustainability education, offering a theoretically grounded and practically actionable model for nursing curriculum development. As healthcare systems worldwide confront the dual imperatives of clinical excellence and environmental responsibility, fostering self-efficacy through high-quality simulation emerges as a strategically powerful approach to cultivating the next generation of sustainability-conscious nursing professionals.

## Figures and Tables

**Table 1:** Demographic characteristics of study participants.

Variable	Category	Psychometric Sample (N = 645)		Main Sample (N = 679)	
		N	%	N	%
Gender	Male	272	42.2	221	32.5
	Female	373	57.8	458	67.5
Age	18	55	8.5	65	9.6
	19	112	17.4	85	12.5
	20	152	23.6	191	28.1
	21	174	27.0	141	20.8
	22	152	23.6	197	29.0
Academic Year	First	185	28.7	177	26.1
	Second	160	24.8	158	23.3
	Third	158	24.5	163	24.0
	Fourth	142	22.0	181	26.7

Note. N = frequency; % = valid percentage of total sample.

**Table 2:** Descriptive statistics for CHEST subscales, SECP subscales, and SANS_2 (main sample, N = 679).

Scale/Subscale	Min	Max	M	SD	Skewness	Kurtosis
Environment and Resources	14.00	25.00	20.64	2.41	0.117	−0.278
Reflect and Support	13.00	25.00	20.98	2.49	−0.138	−0.266
Cultural Relevance	13.00	25.00	20.06	2.55	0.268	−0.217
Skills and Confidence	12.00	25.00	19.73	2.63	0.339	−0.129
Engagement	28.00	50.00	40.27	4.92	0.303	−0.179
Motivation	24.00	50.00	41.27	4.72	0.070	−0.019
CHEST Total	109.00	200.00	163.42	18.87	0.284	−0.110
Assessment	358.00	1200.00	804.80	151.30	−0.044	−0.216
Diagnosis & Planning	200.00	900.00	581.72	113.13	0.044	−0.032
Implementation	342.00	1000.00	698.94	138.50	−0.042	−0.753
Evaluation	188.00	600.00	378.00	72.46	0.121	0.130
SECP Total	1113.00	3700.00	2463.46	464.14	−0.002	−0.323
SANS Total	16.00	35.00	28.12	4.79	−0.155	−0.864

Note. SECP = Self-Efficacy in Clinical Performance Scale; SANS = Sustainability Attitudes in Nursing Survey; M = mean; SD = standard deviation.

**Table 3:** Pearson bivariate correlations among CHEST subscales, SECP subscales, and SANS_2 (main sample, N = 679).

Variable	1	2	3	4	5	6	7	8	9	10	11	12	13
1. Environment and Resources	1												
2. Reflect and Support	0.836 **	1											
3. Cultural Relevance	0.827 **	0.824 **	1										
4. Skills and Confidence	0.825 **	0.829 **	0.840 **	1									
5. Engagement	0.837 **	0.848 **	0.860 **	0.860 **	1								
6. Motivation	0.849 **	0.871 **	0.866 **	0.868 **	0.890 **	1							
7. CHEST Total	0.886 **	0.898 **	0.894 **	0.899 **	0.928 **	0.935 **	1						
8. Assessment	0.676 **	0.725 **	0.674 **	0.686 **	0.697 **	0.714 **	0.721 **	1					
9. Diagnosis & Planning	0.716 **	0.754 **	0.714 **	0.730 **	0.741 **	0.755 **	0.767 **	0.944 **	1				
10. Implementation	0.741 **	0.774 **	0.740 **	0.759 **	0.759 **	0.771 **	0.788 **	0.946 **	0.945 **	1			
11. Evaluation	0.685 **	0.723 **	0.678 **	0.698 **	0.709 **	0.720 **	0.737 **	0.928 **	0.910 **	0.919 **	1		
12. SECP Total	0.723 **	0.764 **	0.721 **	0.737 **	0.745 **	0.759 **	0.772 **	0.983 **	0.976 **	0.981 **	0.955 **	1	
13. SANS Total	0.548 **	0.567 **	0.549 **	0.576 **	0.565 **	0.571 **	0.613 **	0.660 **	0.685 **	0.701 **	0.646 **	0.692 **	1

Note. ** *p* < 0.01 (two-tailed).

**Table 4:** Path coefficients for the mediation model (CHEST → SECP → SANS_2) (main sample, N = 679).

Outcome	Predictor	β*	β	SE	t	*p*	LLCI	ULCI
SANS (Y)	CHEST (X)	0.1943	0.0493	0.010	4.5129	<0.001	0.0278	0.0707
SANS (Y)	SECP (M)	0.5420	0.0056	0.004	12.5858	<0.001	0.0047	0.0065
SECP (M)	CHEST (X)	0.7723	18.9925	0.600	31.6351	<0.001	17.8137	20.1713

Note. β* = standardized coefficient; β = unstandardized coefficient; SE = standard error; LLCI = lower limit confidence interval; ULCI = upper limit confidence interval. Bootstrap 95% CI based on 5000 resamples.

**Table 5:** Total, direct, and indirect effects of CHEST on SANS_2 through SECP (main sample, N = 679).

Effect Type	b	β*	SE	Boot 95% CI	% of Total
Total effect (X → Y)	0.1554	0.6129	0.007	[0.1403, 0.1706]	100.00%
Direct effect (X → Y)	0.0493	0.1943	0.109	[0.0278, 0.0707]	31.73%
Indirect effect via SECP (X → M → Y)	0.1062	0.4186	0.010	[0.0858, 0.1270]	68.34%

## Data Availability

The datasets generated and analyzed during the current study are available from the corresponding authors upon reasonable request.

## Abbreviations

The following abbreviations are used in this manuscript:

CHEST	Comprehensive Healthcare Education Simulation Tool
SECP	Self-Efficacy in Clinical Performance
SANS	Sustainability Attitudes in Nursing Survey
SCT	Social Cognitive Theory
CFA	Confirmatory Factor Analysis
RMSEA	Root Mean Square Error of Approximation
CFI	Comparative Fit Index
GFI	Goodness of Fit Index
CI	Confidence Interval
LLCI	Lower Limit Confidence Interval
ULCI	Upper Limit Confidence Interval
SD	Standard Deviation
SE	Standard Error
SPSS	Statistical Package for the Social Sciences
AMOS	Analysis of Moment Structures
SDGs	Sustainable Development Goals
SBL	Simulation-Based Learning
β*	Standardized Coefficient
R^2^	Coefficient of Determination
